# Technical modelling of solar photovoltaic water pumping system and evaluation of system performance and their socio-economic impact

**DOI:** 10.1016/j.heliyon.2023.e16105

**Published:** 2023-05-08

**Authors:** Salman Habib, Haoming Liu, Muhammad Tamoor, Muhammad Ans Zaka, Youwei Jia, Abdelazim G. Hussien, Hossam M. Zawbaa, Salah Kamel

**Affiliations:** a College of Energy and Electrical Engineering, Hohai University, Nanjing 211100, China; b Department of Electrical and Electronic Engineering, Southern University of Science and Technology, Shenzhen 518055, China; c Department of Electrical Engineering and Technology, Government College University Faisalabad, Faisalabad 38000, Pakistan; d Energy and Environmental Engineer, Zeecon Engineering Services, Faisalabad, Pakistan; e Department of Computer and Information Science, Linköping University, Linköping, Sweden; f Faculty of Science, Fayoum University, Fayoum, Egypt; g MEU Research Unit, Middle East University, Amman, Jordan; h Faculty of Computers and Artificial Intelligence, Beni-Suef University, Beni-Suef 62521, Egypt; i Applied Science Research Center, Applied Science Private University, Amman, Jordan; j Department of Electrical Engineering, Faculty of Engineering, Aswan University, Aswan 81542, Egypt

**Keywords:** Photovoltaic system, Water pumping system, Tilt angle, Variable frequency drive, System losses, Performance ratio, Cost analysis, Socioeconomic impact

## Abstract

Water is a precious resource for agriculture and most of the land is irrigated by tube wells. Diesel engines and electricity-operated pumps are widely used to fulfill irrigation water requirements; such conventional systems are inefficient and costly. With rising concerns about global warming, it is important to choose renewable energy source. In this study, SPVWPS has been optimally designed considering the water requirement, solar resources, tilt angle and orientation, losses in both systems and performance ratio. A PVSyst and SoSiT simulation tools were used to perform simulation analysis of the designed solar photovoltaic WPS. After designing and performance analysis, farmers were interviewed during fieldwork to assess socioeconomic impacts. In the result section, performance of PV system is analyzed at various tilt angles and it is established that system installed at a 15° tilt angle is more efficient. The annual PV array virtual energy at MPP of designed photovoltaic system is 33342 kWh and the annual energy available to operate the WPS is 23502 kWh. Module array mismatch and ohmic wiring losses are 374.16 kWh and 298.83 kWh, respectively. The total annual water demand of the selected site is 80769 m³ and designed SPWPS pumped 75054 m³ of water, supplying 92.93% of the irrigation demand. The normalized values of the effective energy, system losses, collection losses and unused energy in the SPVWP system are 2.6 kW/kWp/day, 0.69 kW/kWp/day, 0.72 kW/kWp/day and 0.48 kW/kWp/day, respectively. The annual average performance ratio of the proposed system is 74.62%. The results of the interviews showed that 70% of farmers are extremely satisfied with the performance of SPVWPS and 84% of farmers indicated that they did not incur any operating costs. The unit cost of the SPWPS is 0.17 €/kWh, which is 56.41% and 19.04% less expensive than the cost of diesel and grid electricity.

## Abbreviations

PV: Photovoltaic
VFD: Variable Frequency Drive
WPS: Water pumping system
SPVWPS: Solar photovoltaic water pumping system
SoSiT: Solar-Drip Simulation Tool
Kc: Crop coefficient
CWR: Crop water requirements
Eto: Reference evapotranspiration
STC: Standard test conditions
NOCT: Nominal operating cell temperature
PVAC: PV array configuration
Imp: Current at maximum power
Vmp: Voltage at maximum power
GHI: Global horizontal irradiance
DHI: Diffuse horizontal irradiance
kWh: kilowatt-hour
kW: kilowatt
MW: Megawatt
LPS: Liter per second
PR: Performance ratio
CBM: Cubic meter
AC: Alternating current
DC: Direct current
MPPT: Maximum power point tracking
Isc: Short circuit current
Voc: Voltage at open circuit

## 1 Introduction

The most environmental friendly and safe option among different renewable resources is solar photovoltaic (PV) energy [1]. Pakistan is rich in solar energy that can be used anywhere for a variety of applications. Pakistan receives nearly 15.53 10^14^ kWh/m^2^ of solar irradiance per year, and there are approximately 7.0 to 9.0 h of sunshine every day [2]. Solar photovoltaic (SPV) cells convert the sun irradiance into electrical energy. Large utility scale energy generation systems, solar home systems, water pumping system (WPS), spacecraft, satellites and the reverse osmosis (RO) plants are important applications of solar photovoltaic cells [3,4]. The most popular types of solar photovoltaic systems are off-grid systems, grid-tied systems and hybrid systems [5]. SPV water pumping system (WPS) is one of the most efficient and best solutions for providing water in remote areas that have limited or no access to national power distribution system [6,7].

Agricultural activities in developing countries are extremely dependent on rainfall. In these countries, the national government and regional governments have taken a number of steps, such as rain water harvesting, ponds, proper construction and maintenance of lakes, and construction of large dams across rivers, etc. to increase the availability of surface and ground water for agriculture purposes throughout the year. Pumps are used to supply water to fields in the majority of locations [8]. Pumping water is a fundamental function in agriculture because adequate and timely water supplies enhance the crop yields. The majority of farmers rely on groundwater for their agricultural operations [9], and in different areas of Africa and Asia, buckets are commonly utilized to irrigate the crops, resulting in a decline in crop yields [10]. Electricity from utility grid or diesel generators are typically used to pump water in agricultural sector. However, further development of these systems is hinder by increasing fossil fuel prices, carbon dioxide emissions, and unreliable utility grid and infrastructure in rural and arid areas [11]. In the agricultural sector, Pakistan seems to have more than 600,000 water tube wells. These pumps use a significant amount of energy to operate. A number of pumps powered by electricity from utility grid or diesel engine, are utilized to extract the water from the ground [12,13].

Utilizing renewable energy for water pumping is one best proposed method for making agriculture economical and sustainable [14]. Solar (PV) energy [15], wind energy [16], and biogas energy [17] are the three potential renewable energy systems that could be used for WPS. The usage of photovoltaic technology has the potential to be expanded, and it also provides an environmental friendly alternative energy for water pumps to operate that previously use electricity from fossil fuel (diesel, natural gas etc.) [18,19]. Furthermore, the significance of photovoltaic system to drive water pumps has grown due to continued depletions of fossil fuel sources and rising energy prices, which is primary area of concern for developing countries [20]. Crops need a huge amount of water during sunny day or throughout months. Since there is a natural correlation between crop water demand and availability of solar energy. Therefore, solar energy is most suitable and best RES for the water pumping in agriculture sector because its period of availability matches the time when crops demand more water. Furthermore, solar photovoltaic WPS is a more practical and reliable option for irrigating agriculture lands in rural or arid areas, because these areas are not connected to utility power network in many countries [21,22].

Photovoltaic energy systems are gaining considerable attention from researchers and policymakers as a feasible and suitable alternative for conventional energy systems to operate water pumping system in agriculture sector [23]. The photovoltaic power generation have demonstrated remarkable environmental and economic performance when compared to diesel engines powered irrigation systems [24] in both urban and rural regions, with a payback period of 4 to 5 years [25]. Irrigation (water pumps) is one of the most appropriate applications of photovoltaic energy that have become more prevalent in recent years [26,27]. This energy generating technology is appealing to many agricultural producers or farmers because it significantly enhances their income by reducing their fuel or energy expenses [[28], [29], [30]]. Additionally, energy produced by a photovoltaic system is used efficiently to perform the other operations in the farm during the off-season when irrigation water is not needed [31,32].

Sontake and Kalamkar provided an overview of the research on several techniques for improving solar photovoltaic WPS performance and optimize PV array size in various climatic regions of the world [33]. The performance of centrifugal deep well pumps with different PV array configurations (PVAC) at pumping heads of 2, 3, and 4 bars was studied under outdoor conditions in India to select the optimum PV array configuration. It has been found that, (4 series x 2 parallel) PV array configuration (PVAC) provided the required discharge at the lowest possible cost whereas (5 series x 2 parallel) PVAC produced maximum discharge at all heads [34]. Buthigg et al. [35] examined performance of the four SPVWPS using the four different photovoltaic array configurations in Ghardaia, Algeria. After experiment they concluded that (2 series x 1 parallel) PV array configuration (PVAC) has highest efficiency as compared to other PVAC. Muhsen et al. [36] suggested a multi objective iterative algorithm to choose the optimum PV array configuration that could balance the cost, reliability and additional water of PV water pumping system. Tiwari and Kalamkar [37] studied the performance of solar photovoltaic WPS under outdoor conditions in India, using helical pumps operated by different PV array configurations.

The performance of photovoltaic array is dependent on ambient temperature, intensity of solar irradiation, and the surface temperatures on the both sides (bottom and top) of modules [38]. These factors fluctuate throughout the year for a particular region depending on the season [39]. These seasonal fluctuations are considering while sizing and designing the major components of water pumping system (WPS). Mokeddem et al. [40] examined the performance of directly coupled solar photovoltaic WPS at 6.0 and 11.0 m static head under different solar irradiance levels in Algeria. Benghanem et al. [41] studied the performance of solar photovoltaic WPS have DC helical by varying solar irradiation and pump head in Saudi Arabia. The output energy and performance variations of a photovoltaic systems with changes in temperature and season was examined by Zaghba et al. [42]. They concluded that the output energy of the photovoltaic modules varies nonlinearly at lower irradiation intensity and linearly at higher irradiation intensity.

Recent literature studies have shown that the use of photovoltaic water pumping system is sustainable, efficient and cost effective. In addition, the literature also highlights the technical feasibility, reliability and bi-directional capability of SPVWPS [43,44]. Solar photovoltaic WPS is the optimal and ideal alternative to utility grid and diesel engine operated water pumps as it offers exceptional socio-economic and environmental features [45]. Solar photovoltaic water pumping system offers number of advantages over petrol or diesel engine operated water pumps. The environmental advantages are nearly zero pollutant emissions, no fuel requirements, and low noise. Furthermore, the cost of SPVWPS is much lower and shorter payback period [46,47]. The assessment of different types of power converters that are utilized in solar PV water pumping system have been examined in References [[48], [49], [50]]. The function of soft computing methodologies for modelling the photovoltaic systems with software like PVSyst, PVGIS, PV Sol, etc., from which the optimal PV system for water pumping system can be chosen have been discussed in Reference [[51], [52], [53]]. The designing, simulation modelling and performance analysis of SPVWP systems have been discussed in References [47,54]. The comparative analysis of the PVSyst simulation software with other simulation software for photovoltaic system is shown in the Table 1.

**Table 1:** Comparative analysis of PVSyst with other photovoltaic system simulation software [39].

	PVSyst	HelioScope	PV*SOL	SAM
Modelling time step	Hourly	Hourly	Hourly	Hourly
Model of PV Module	Single diode (Shockley's model)	Single diode (Shockley's model)	Enhances single diode	Single diode (CEC model)
Decomposition GHI	Erbs Model	Erbs Model	Reindl	N/A
Albedo	0.2	0.2	0.2	0.2
Temperature Model	Thermal balance equation	Sandia Model	Thermal balance equation	NOCT
Transposition Model	Perez Model and Hay's Model	Perez Model	Hay-Davies	Perez Model
Irradiation components	User selection	GHI and DHI	GHI	DNI and DHI
Incidence Angle Modifier loss/Module cover	Model dependent (ASHRAE for old & Fresnel for new modules)	ASHRAE	ASHRAE	Model dependent
AC cabling/Wiring losses	0%	0.50%	N/A	1%
Losses due to Module Mismatch	1%	Calculated	2%	2%
DC wiring Losses	1.50%	Calculated	Calculated	2%
Pump Simulation	✓	×	✓	×

The price of photovoltaic (PV) modules has recently decreased in all over the world. Although, initial capital cost of the SPV water pumping system (WPS) are still high as compared to conventional water pumping system operated by electricity from national grid or diesel engine. The initial capital cost of SPVWPS are considered as a primary challenge to the use of this pumping system in developing countries. In this context, the main objective of this research study is to develop an optimized model and performance analysis of PV system and solar photovoltaic WPS for rural or remote areas that face serious energy crises. The primary contributions of this research are assessment of solar and water resources, and daily water requirements for the selected site. Modelling of a photovoltaic system and solar photovoltaic WPS to fulfil water demand. Comparative analysis of output energy produced by the PV system installed with fixed-mounted structure and single-axis solar tracker, and finding the optimum tilt angle for the photovoltaic array. A PVSyst and SoSiT simulation tools are used to perform simulation analysis of the designed solar photovoltaic WPS. Estimations of energy losses in PV systems and pumping systems. The performance of photovoltaic module is analyzed at different temperature levels, and the relationship between power (W) available to operate the pumping system and flow rate (m^3^/h) as well as daily water productions with effective global irradiation are analyzed. A cost analysis is also performed to compare SPVWPS with conventional pumping systems (diesel engine and utility grid). After this, farmers are interviewed during fieldwork to assess socioeconomic impacts of solar photovoltaic WPS.

Remaining sections of the research study are structured as: Section 2--Materials and Methods, that includes mathematical model of the SPVWPS, designing of photovoltaic system, system components, pumping system and water resource details. Section 3--Results, this section discusses solar resources at the study area, performance analysis of PV system at different tilt angle, losses in the photovoltaic system, performance analysis of SPVWPS, experimental results, monthly variation in performance ratio (PR), relationship between available energy and pump flow rate, daily water production versus effective global irradiation and losses in the pumping system. A cost analysis is explained in the Section--4. Discussion and conclusions of research study is discussed in Section—5.

## 2 Materials and methods

In this study, a promising agricultural site (latitude 24.90°, longitude 67.00°) in Pakistan was chosen to analyze the performance of a solar photovoltaic WPS. The seasonal weather pattern varies from cold winters to sunny summers with peak temperature of 31.09 °C. Approximately 60–75% of the annual rainfall occurs during the monsoon season from month of July to September and there are an average 7 to 9 sunshine hours per day. In the proposed location, the average wind velocity is less than 3.6 m/s. The average monthly ambient temperature is 26.88 °C. In accordance with the data, a photovoltaic (PV) system can capture a sufficient amount of sustainable solar energy. Solar irradiation data of the proposed location was taken from the Meteonorm database and then imported into the PVSyst simulation database by using its meteorological tools. Meteonorm is a novel combination of reliable data sources with advanced computation techniques. It gives users access to historical time series and typical years. For any location on earth, Meteonorm software primarily gives hourly and monthly meteorological data. More than 30 different meteorological parameters were accessible to researchers i.e. GHI, DHI, wind speed and temperature etc. A globally calibrated aerosol-climatology, five geostationary satellites, and more than 8000.0 weather stations constitute the data base [55].

**Fig. 1:**
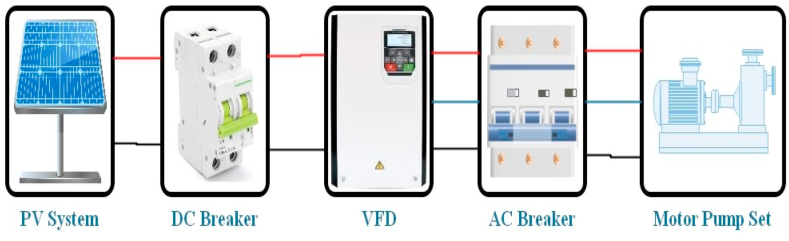
Experimental setup for solar photovoltaic WPS.

The experimental setup for solar photovoltaic WPS is shown in Fig. 1. It consists of photovoltaic system, variable-frequency drive (VFD), AC/DC breakers, an AC induction motor (three-phase), and the centrifugal water pump (either surface mounted or submersible). Three-phase AC induction motor is commonly coupled water pumping system in agriculture sector due to their simple maintenance, operation and the provision of AC power from the national utility grid. A VFD must be installed to convert DC power of PV system into AC power because AC motors are not be connected directly to the photovoltaic array. A variable frequency drive (VFD) also known as solar pump inverter that convert DC power of the PV array into AC Power. A VFD drives an electric motor by varying the voltage and frequency of its power supply. The motor's ramp-up and ramp-down during start and stop, respectively, can also be controlled by the VFD. It can increase efficiency and reduce production losses.

### 2.1 Sizing of the solar photovoltaic WPS

Under-sizing will result in unsatisfactory system performance, while oversizing would result in excessive costs. To meet the particular needs of the site, each system component must be designed and sized in an optimal way. It is the only way to achieve the expected performance while simultaneously ensuring system stability, durability, and sustainability. The procedures that need to be followed in order to size a water pumping system that is powered by a photovoltaic system are water resource assessment, total head, water demand, required flowrate, assessment of solar resources, sizing of PV system and water pump.

### 2.2 Crops water requirement

There are seven zones at specific solar photovoltaic WPS site. Each zone have area of (4 acres) and these zones are designated for row crops. The maximum crop water requirement is 7750 m^3^/h per month in June and minimum crop water requirement is 2196 m^3^/h per month in December as shown in Table 2. The impact of different climatic conditions is included in Eto and crop attributes/characteristics in the crop coefficient (Kc). Reference evapotranspiration (Eto) is the quantity of water required by a specific crop or plant. Crop coefficient (Kc) is ratio of actual crop evapotranspiration to the crop reference evapotranspiration. Canopy factor is calculated by dividing the wetted area by canopy area. It frequently varies between 0.4 for crops with medium spacing and 0.3 for widely spaced crops. Due to many causes of water loss, the amount of supplied water is not fully used for the crops growth. The ratio of the amount of water used to fulfil the crop's consumptive demands that needed to maintain a favorable water balance in the root of crop to the total volume of water pumped, channeled or stored for irrigation is known as irrigation efficiency.

**Table 2:** Row crops water requirements.

	Jan	Feb	Mar	Apr	May	Jun	Jul	Aug	Sep	Oct	Nov	Dec
Eto (mm/day)	3.61	4.45	6.6	6.97	7.2	6.97	6.4	7.22	6.97	7.23	3.27	2.04
Kc	0.7	0.7	0.7	0.7	0.7	0.7	0.7	0.7	0.7	0.7	0.7	0.7
Crop water requirements (mm/day)	2.5	3.1	4.6	4.9	5.0	4.9	4.5	5.1	4.9	5.1	2.3	1.4
Irrigation Efficiency %	70%	70%	70%	70%	70%	70%	70%	70%	70%	70%	70%	70%
Canopy factor	0.4	0.4	0.4	0.4	0.4	0.4	0.4	0.4	0.4	0.4	0.4	0.4
Application Rate mm/hour	3.98	3.98	3.98	3.98	3.98	3.98	3.98	3.98	3.98	3.98	3.98	3.98
No. of Zones	7.0	7.0	7.0	7.0	7.0	7.0	7.0	7.0	7.0	7.0	7.0	7.0
Operation time (hrs/day)	0.4	0.4	0.7	0.7	0.7	0.7	0.6	0.7	0.7	0.7	0.3	0.2
Total operational time hours	2.5	3.1	4.6	4.9	5.1	4.9	4.5	5.1	4.9	5.1	2.3	1.4
Flow rate (lps)	14.16	14.16	14.16	14.16	14.16	14.16	14.16	14.16	14.16	14.16	14.16	14.16
Monthly volume requirement (m^3^/h)	3890	4782	7107	7500	7750	7500	6883	7570	7500	7573	3522	2196

### 2.3 Mathematical modelling

The mathematical model of solar photovoltaic (PV) WPS comprises calculations of pump hydraulic power, motor power, photovoltaic array sizing and system configurations.

#### 2.3.1 Water pump hydraulic power

Hydraulic power of water pumping system depends on the designed head (H in meter) and water flow rate (Q in m^3^/h) of the system. The required hydraulic power of the pumping system is computed by using equation (1).

(1) $$P_{H}\left(kW\right)=\frac{H\times Q\times \rho \times g}{3.6\times 10^{6}}$$

Where

*g* : Gravitational acceleration (9.81 m/s^2^)
*ρ* : Water density in kilogram/m^3^,

The hydraulic head of pumping system consisting of three primary terms [56,57] as shown in equation (2).

(2) $$H=H_{s}+H_{dd}+H_{f}$$

Static head is represented by $H_{s}$ and it is the difference between groundwater level and water level at the discharge point. Dawdown water level is represented by $H_{dd}$, while friction losses in the hydraulic circuit are represented by $H_{f}$. In this research study, the both drawdown water level and static head $\left(H_{dd}+H_{s}\right)$ is 55 m. Frictional losses are 0.100, in piping as a result of fitting parts like valves, elbows, pipe junctions, and fluid viscosity. So total head is 60.5, flow rate is 51 m^3^/h.

$$P_{H}\left(kW\right)=\frac{60.5\times 51\times 1000\times 9.8}{3.6\times 10^{6}}$$

$$P_{H}\left(kW\right)=8.39kW$$

#### 2.3.2 Calculation of motor power

Both surface and submersible water pumps are normally installed for irrigation purposes in agriculture sector. These water pumps are operated by three-phase AC induction motors. These motor-pump set are selected primarily due to their high efficiency and potential to deliver the required flow rate for specific head. The power required by motor is calculated by equation (3) and depend on the efficiency of the water pump.

(3) $$Power\;Required\;by\;the\;Motor=\frac{Hydraulic\;Power\;Required\;by\;Water\;Pump}{Efficiency\;of\;the\;Water\;Pump}$$

$$Power\;Required\;by\;the\;Motor=\frac{8.39}{53}\times 100$$

PowerRequiredbytheMotor=15.84kW

#### 2.3.3 Photovoltaic array sizing

For solar photovoltaic WPS, the photovoltaic system depends on the power required by induction motor, system efficiency and daily water requirement. The photovoltaic power requirement for the water pumping system is calculated by using equation (4):

(4) $$Total\;Power\;from\;PV\;System\;\left(kW\right)=\frac{Power\;Required\;by\;the\;Motor}{Efficiency\;of\;the\;System}$$

$$Total\;Power\;from\;PV\;System\;\left(kW\right)=\frac{15.84}{70.5}\times 100$$

TotalPowerfromPVSystem(kW)=22.47kW

#### 2.3.4 System configurations

Two configurations of solar photovoltaic WPS are used in agriculture sector (i) direct coupling and (ii) coupling by using VFD. Due to significant technical issues, direct coupling configuration is not prominent in the agricultural sector. This study examines configurations of s solar photovoltaic WPS using VFD have a maximum power point tracking (MPPT) function.

### 2.4 Designing of SPV water pumping system

#### 2.4.1 Designing of photovoltaic system

To achieve an optimal PV output energy, it is critical and important to make the proper selection of PV modules [58,59]. As a result, it will reduce installation, maintenance and overall system cost. For large scale power systems, it is recommended to select photovoltaic modules with higher efficiency and wattage. ABi-Solar (AB450-72MHC) 450-W Si-mono HC PV modules and VEICHI variable frequency drive are used in this system. The specification of ABi-Solar (AB450-72MHC) are shown in Table 3.

**Table 3:** ABi-Solar (AB450-72MHC) specification.

Description	Value
Maximum Power of Module (P_max_)	450 W
Short Circuit Current (I_sc_)	11.470 A
Maxi. Power Current (I_mpp_)	10.880 A
Maxi. Power Voltage (V_mpp_)	41.400 V
Open-Circuit Voltage of module (V_oc_)	50.00 V
PV Module Efficiency	20.4%
Maxi. System Voltage	1000V VDC
Maxi. Series Fuse	20A

Variable frequency drive is basically a motor drive used in electromechanical drive systems to control torque and speed of alternating current (AC) motors by varying the input frequency and also adjust the current or voltage variation [60]. The specifications of VFD is shown in the Table 4 and design of photovoltaic energy generation system is shown in Table 5.

**Table 4:** Specifications of VFD.

Description	Value	Description	Value
Min. Voltage at MPP	284 V	Max. Voltage at MPP	519 V
Max. PV Array Voltage	629 V	Max. Input Current	63.9 A
Max. efficiency	97.0%	EURO efficiency	95.0%

**Table 5:** Design of PV system.

Number of Photovoltaic (PV) Modules			
PV Modules in series	11.0	No. of strings	5.0
Total Photovoltaic modules	55	Module maximum power	450 Watt
PV array power (STC))	24.75 kW	PV array power (NTC)	22.84 kW
Operating Characteristics of PV Array			
U mpp	425 V	I mpp	54 A
Collector Plane Orientation			
Tilt Angle	15°	Azimuth	180°
Frame size	8 × 2 Single Axis Tracking	Orientation	Landscape

**Fig. 2:**
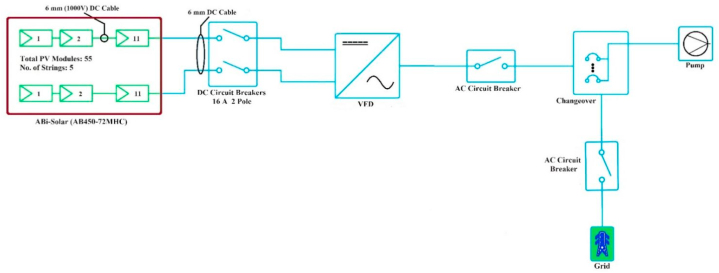
SLD of Solar Photovoltaic system for WPS.

Single line diagram (SLD) of a photovoltaic system that used operate water pumping systems is shown in Fig. 2. As seen from the SLD, the VEICHI VFD is connected to five strings, each of which have 11 photovoltaic modules (AB450-72MHC). A successful system installation depends on the wiring configuration because it reduces system loss and enhances operational safety. With 6 mm (1000V) single core DC cable, the photovoltaic modules are connected in string. Two AC circuit breakers (3 Pole, 32 A AC) and one DC circuit breaker (2 Pole, 16 A DC) are connected. The first AC breaker is used to disconnect the VFD and the second AC breaker is used to disconnect the utility grid.

#### 2.4.2 Pumping system and water resource details

Table 6 summarizes the parameters for the pumping system and water resource.

**Table 6:** Parameters for the pumping system and water resource.

**Well parameters**			
Static depth level	55 m	Specific drawdown	0.00 m/m³/hour
Water pump depth	110 m	Maximum depth of water pumping	92 m
Water storage tank			
Volume	1000.0 m³	Diameter	8.1 m
Feeding altitude	5.0 m	Water full height	19.2 m
Hydraulic circuit			
Water piping length	152 m	Friction losses	0.100

#### 2.4.3 Solar pump details

Table 7 presents about details of solar pump installed in solar photovoltaic WPS.

**Table 7:** Solar Pump details.

Description	Value
Manufacturer	Grundfos
Model	SP 95-4
Pump Technology	Centrifugal multistage
Motor Type	AC motor, triphased
Running Head	27 m Head Min, 55.0 m Head Nom and 82.0 m Head Max
Nominal Voltage	400 V tri

Table 8 shows the flow rate of a water pump connected with a photovoltaic system. As seen from the table, the flow rate the water pump increases as the output power (kW) of the photovoltaic power increases. When the output power of the PV system is less than 6 kW, the flow rate is zero. With an increase in output power, the flow rate gradually increases. The required flow rate of the pump is achieved with 17 kW of PV power.

**Table 8:** Output flow points of installed Pump.

PV System (kW)	Output Flowrate of Pump		PV System (kW)	Output Flowrate of Pump	
	LPS	m^3^/h		LPS	m^3^/h
0.00	0.00	0.00	11.00	8.56	30.816
1.00	0.00	0.00	12.00	9.44	33.984
2.00	0.00	0.00	13.00	10.56	38.016
3.00	0.00	0.00	14.00	11.33	40.788
4.00	0.00	0.00	15.00	12.17	43.812
5.00	0.00	0.00	16.00	13.10	47.16
6.00	1.72	6.192	17.00	14.16	50.99
7.00	3.39	12.204	18.00	14.51	52.236
8.00	4.78	17.208	19.00	14.51	52.236
9.00	6.17	22.212	20.00	14.51	52.236
10.00	7.28	26.208			

**Fig. 3:**
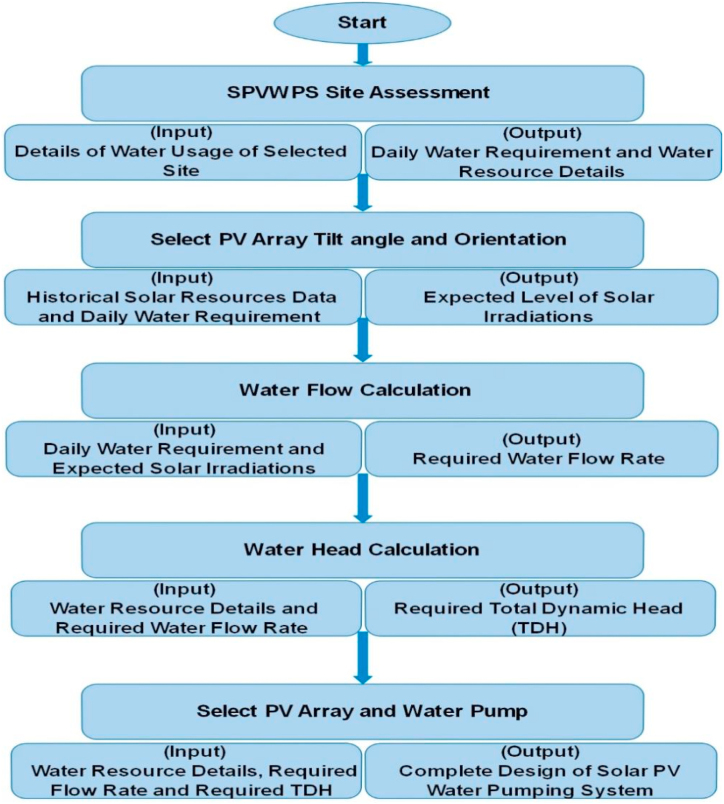
Flowchart for designing of solar PV water pumping system.

The flowchart for designing of solar photovoltaic WPS is shown in Fig. 3. This flowchart shows all the main steps involved in designing of a solar photovoltaic WPS starting from the site assessment.

### 2.5 Systems simulation

In order to examine the pattern of energy generation, the overall amount of generated energy, and to determine the possible amount of available water, the system is simulate according to the selected technical design. These results are compared to the amount of water needed for irrigation. Based on factors including the selected PV design, water pump, irradiance, temperatures and under/over watering, the findings from the simulation are provides an estimate of the output energy potential from PV system and potential output water from pumping system.

#### 2.5.1 PVSyst

PVSyst is a useful and effective simulation software for designing solar photovoltaic (PV) systems. The PVSyst simulation software provides many features, including system design, system sizing (which includes sizing of photovoltaic array and VFD), simulation and results, system component database, pricing strategy (feed-in tariff etc). During the designing of solar photovoltaic WPS, simulations were performed based on the maximum possible annual water demand assuming that PV modules are not shaded. PV modules and water pumps are selected from the database of PVsyst simulation software to fulfil the maximum possible annual demand.

#### 2.5.2 SoSiT

SoSiT [47] simulation tool incorporates the features of a PVSyst [61] that facilitates in the designing of a solar photovoltaic WPS and demonstrates how various factors influence the system performance, and CropWat [62] evaluates crop water requirements, that are crucial for irrigation. SoSit simulation tool is based on one-year hourly analysis (8760.0 h). Input parameters like solar irradiance, ambient temperature and water needs are calculated for each hour. These parameters are used to calculate output values, such as net photovoltaic system energy generation and the resulting output water by the irrigation system.

The other key input parameters of the SoSiT approach are: the size of PV system in kWp, monthly water requirement for the crops, site location, and the output flow points of installed Motor/Pump combination at various levels of input power levels. As a result, the SoSiT simulation tool calculates all decision variables for every hour of five years like expected output water of system, annual overcapacity/shortfall of the system, maximum weekly and monthly supply gap, annually water supply gap (CBM) and the water efficiency (CBM/kWh).

### 2.6 PV structures

**Fig. 4:**
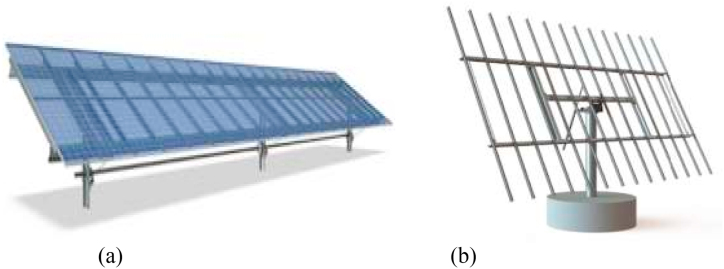
PV structures (a) Fixed-mounted structure (b) single-axis solar tracker.

The basic design for structure of photovoltaic (PV) power plants can be divided into two categories: tracked systems and fixed systems. In tracked systems, the PV modules are mounted on a moving structure that moves along one or both axes to track the sun during the day. In fixed systems, the PV modules are mounted on a fixed structure. A tracked system has a better yield for a same installed capacity, whereas a fixed structure has cheaper cost and maintenance. This study aims to compare the output energy produced by the PV system installed with fixed mounted structure and single axis solar tracker. With the same installed capacity of 15.3 kWp, simulation using PVSyst are performed. The fixed mounted structure is shown in Fig. 4(a) and single axis solar tracker is shown in Fig. 4(b).

### 2.7 Socio-economic impact

In order to collect reliable data for the research, a survey is conducted of agri. farms that have photovoltaic operated WPS for the past 6 months. The primary data for the research is collected using a questionnaire that included information about the irrigation methods, sources of energy and water, cropping patterns, input costs, and income. For the purpose of collecting data from 20 agriculture farms, a comprehensive on-site survey was carried out. Survey data collected survey was analyzed to present the findings in this research.

## 3 Results

### 3.1 Solar resources

**Fig. 5:**
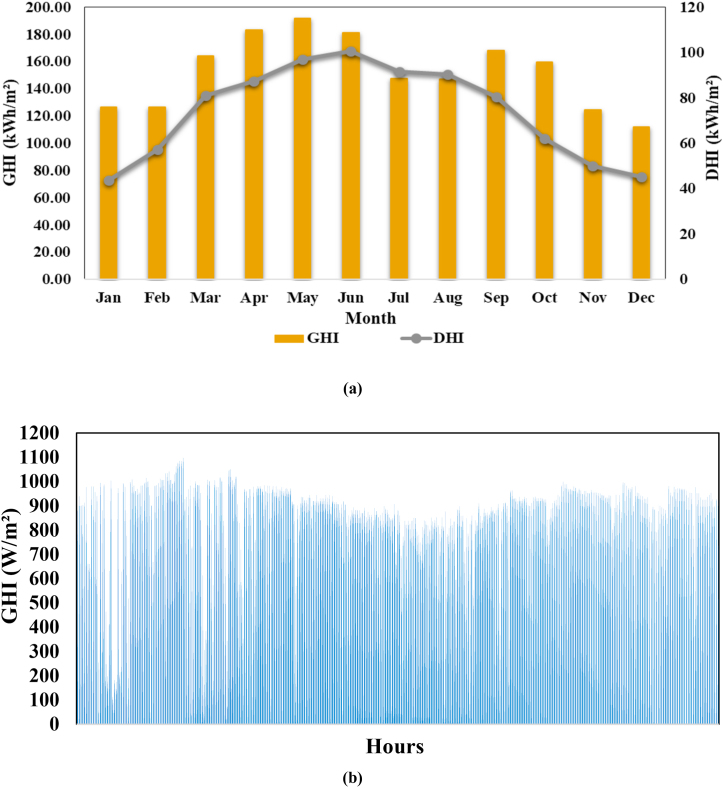
Solar irradiation (a) Monthly variations in DHI and GHI (b) Hourly variations in GHI.

Fig. 5(a) shows that the global horizontal irradiance (GHI) is maximum during the summer season (April to June) and decreases during the winter season (November to February) and Fig. 5(b) shows the hourly variations in GHI.

**Fig. 6:**
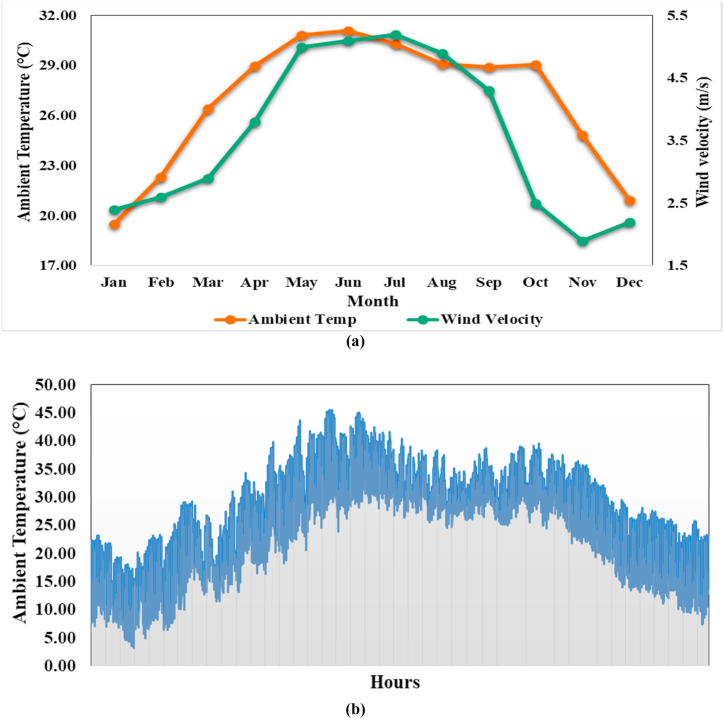
(a) Monthly variations in wind velocity and ambient temperatures (b) hourly variations in ambient temperatures.

The maximum GHI are in month of May (191.7 kWh/m^2^) and minimum in month of December (112.3 kWh/m^2^). The diffuse horizontal irradiance (DHI) is maximum in month of June (100.8 kWh/m^2^) and minimum in month January (43.7 kWh/m^2^). Based on global horizontal irradiance data, not only photovoltaic water pumping system, but many other PV system are installed to fulfil the growing energy demand. The monthly variations in the wind velocity (m/s) and ambient temperatures (°C) at the study site are shown in Fig. 6(a) and hourly variations in ambient temperatures (°C) is shown in Fig. 6(b).

Fig. 4 shows the monthly variations in the wind velocity (m/s) and ambient temperature (°C) at the selected site. Average peak ambient temperatures of the site are in the range of 29–32 °C, during the summer season (May to August) and the wind velocity during this period are in range of 4.5–5.5 m/s. Average monthly ambient temperature of the site is maximum in month of Jun (31.09 °C) with average wind velocity of 5.1 m/s. On the other hand, lower wind velocity and ambient temperatures were measured for the rest of the year. According to Ref. [38], photovoltaic energy generation systems are suitable for the environments where the annual ambient temperature is around 25 °C. For the selected research site, the annual average ambient temperature (°C) was measured to be 26.88 °C. In a technical sense, this indicates that the outdoor climatic conditions in this region are favorable for PV-WPS.

### 3.2 MPPT performance of photovoltaic module

**Fig. 7:**
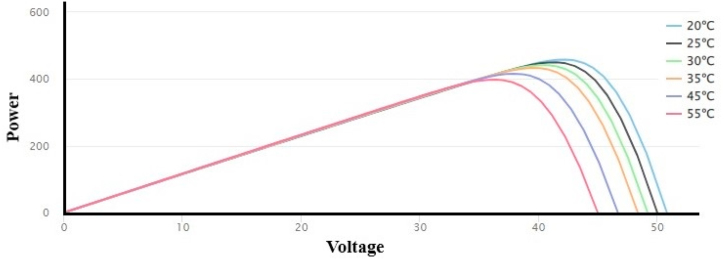
Power-voltage (P–V) at various temperature levels.

Table 9 presents the performance of the selected PV module ABi-Solar (AB450-72MHC) at different temperatures like (25 °C, 30 °C, 35 °C, 45 °C and 55 °C) and at 1000.0 W/m2 irradiance level. The power-voltage (P–V) curve in Fig. 7 depicts the relationship between the power and voltage of the PV modules at various temperature levels.

**Table 9:** PV module output power variations at different temperature levels.

Temperature (°C)	I_SC_	V_OC_	I_MP_	V_MP_	Power	dP_mp_/dT	dV_mp_/dT	dV_oc_/dT
25	11.54	50.0	10.90	41.3	450.1	−0.37%	−0.40%	−0.33%
30	11.57	49.2	10.92	40.4	441.7	−0.38%	−0.41%	−0.33%
35	11.61	48.4	10.94	39.6	433.2	−0.39%	−0.42%	−0.34%
45	11.67	46.7	10.96	37.9	415.9	−0.42%	−0.45%	−0.36%
55	11.74	45.0	10.99	36.2	398.5	−0.45%	−0.47%	−0.38%

The results obtained shows that there is an indirect relationship between output power and temperature. It has been observed that the efficiency of photovoltaic modules reduces as their temperature increases. Table 9 shows that the output power of the photovoltaic module is 450.1 W at operating temperature of 25 °C and an solar irradiance level of 1000 W/m^2^, and the output power is 398.5 W at operating temperature of 50 °C and an solar irradiance level of 1000 W/m^2^.

### 3.3 Performance analysis of PV system installed with different mounting structures

**Fig. 8:**
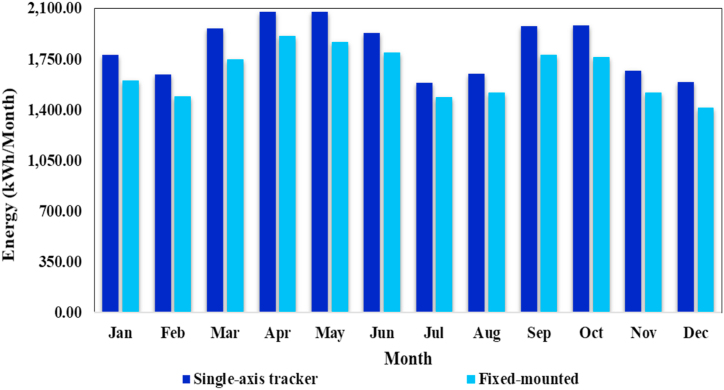
Performance analysis of photovoltaic system installed with different mounting structures.

This study aims to compare the output energy produced by the PV system installed with fixed mounted structure and single axis solar tracker. With the same installed capacity of 15.3 kWp, simulation using PVSyst are performed. Fig. 8 shows an assessment of the monthly energy production from PV systems installed with fixed-mounted structure and single-axis solar tracker, which would help to determine the optimal structure type for maximum power generation of the PV system.

As seen from the figure, the PV system installed with single-axis solar tracker generate more energy as compared to the PV system installed with fixed-mounted structure. In comparison to fixed-mounted structure, single-axis has been shown to be very efficient in terms of output energy. Compared to fixed installations, single-axis solar tracker works effectively even on cloudy and foggy days. As a result, single-axis tracking systems are more efficient than fixed-mounted structure. Although the single axis tracker has more complex hardware, but it offers higher efficiency/performance and is more economical.

### 3.4 Performance analysis of photovoltaic system at different tilt angles

**Fig. 9:**
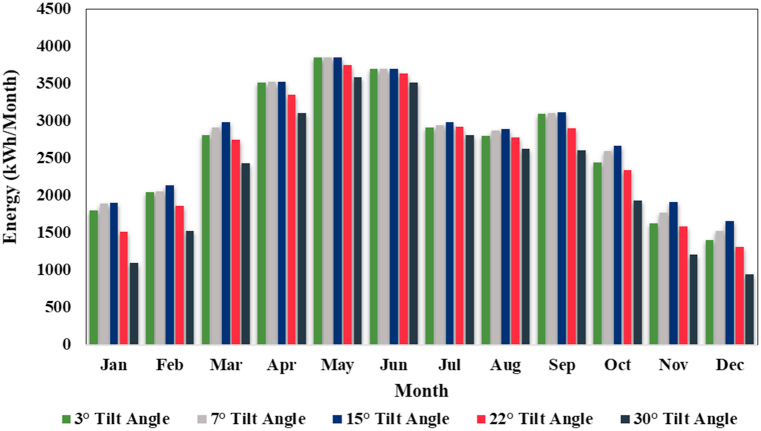
Comparative analysis of monthly energy generation at different tilt angles.

In this research work, a series of simulation experiments are performed in order to determine the optimum tilt angle for the photovoltaic array, which is the angle at which the PV system (24.75 kW designed for WPS) installed with single axis solar tracker produces the maximum output energy at the proposed location. The best strategy/method to maximize the amount of output energy (kWh) from photovoltaic array is to install the array at the ideal tilt angle [39]. Fig. 9 shows an assessment of the monthly energy production from PV systems installed at different tilt degrees (3° to 30°), which helps in determining the optimal tilt angle.

After evaluating the experimental results, it can be determined that photovoltaic (PV) energy production system installed at a tilt angle of 15° is more efficient as compared to photovoltaic systems installed at other tilt angles. The annual generation of PV system is 33342 kWh at this tilt angle, while the annual energy generation at 3°, 7°, 22° and 30° are 32030 kWh, 32771 kWh, 30738 kWh and 27418 kWh respectively. The maximum output energy produced in June (3849 kWh) and the minimum output energy produced in December (1658 kWh). In the simulation results, system losses such as module array mismatch losses, ohmic wiring losses are calculated as shown in Table 10. For a PV energy system installed at a 15° angle, the energy wasted/consumed due to module array mismatch losses and ohmic wiring losses are 374.16 kWh and 298.83 kWh respectively.

**Table 10:** Losses in the Photovoltaic system.

	Losses due to PV Array Mismatch	Losses due to Ohmic Wiring	PV Array Virtual Energy at Maximum Power Point (MPP)	PV Array Virtual Energy at Fixed Voltage	Effective Output Energy of PV Array
	kWh	kWh	kWh	kWh	kWh
January	21.29	11.73	1902	1907	1902
February	23.95	15.78	2137	2143	2137
March	33.52	26.79	2987	2990	2987
April	39.63	36.40	3527	3537	3525
May	43.27	41.42	3849	3850	3849
June	41.58	38.48	3700	3702	3700
July	33.50	27.69	2984	2988	2984
August	32.47	27.00	2893	2901	2892
September	35.01	29.47	3118	3120	3117
October	29.94	22.30	2669	2674	2669
November	21.46	12.56	1917	1923	1916
December	18.54	9.19	1658	1667	1658
Year	374.16	298.83	33342	33400	33335

### 3.5 Performance analysis of SPVWPS

#### 3.5.1 Balance simulation results of SPVWPS

The main and balances simulation results of the designed solar photovoltaic WPS for the selected site are shown in Table 11. The annual photovoltaic array virtual energy at MPP of the designed photovoltaic system is 33342 kW-hour and the annual energy available to operate the WPS is 23502 kW-hours. The efficiency of PV system is 70.50%. The total annual water demand of the site is 80769 m³ and the total water pumped volume is 73603 m³.

**Table 11:** Simulation results of SPVWPS.

	PV array virtual energy at maximum power point (kWh)	Energy to operate pumping system (kWh)	Unused energy (kWh)	Average total head at water pump (meterW)	Water pumped volume (m³)	Water drawn by farmer (m³)	Missing water (m³)
January	1902	1381	0	60.74	3890	3880	1830
February	2137	1703	0	61.19	4873	4782	1711
March	2987	2443	42	61.83	7943	7117	0
April	3527	2263	857	62.12	7500	7500	0
May	3849	2209	1055	62.50	7782	7750	0
June	3700	2180	948	62.20	7344	7500	0
July	2984	2049	220	62.04	6900	6883	0
August	2893	2266	49	62.11	7580	7570	0
September	3118	2369	256	62.07	7917	7500	0
October	2669	2142	22	61.51	7580	7573	150
November	1917	1398	0	60.80	3549	3522	1768
December	1658	1097	0	60.47	2196	2196	1537
Year	33342	23502	3448	61.69	75054	73773	6996

Based on the simulation results shown in Table 11, the designed solar photovoltaic water pumping system can meet 92.93% of the irrigation water demand of the selected site. This system efficiency is better than that in the study (81.6%) conducted by Mishra et al. [63]. The SPVWPS fulfil the complete water demands in summer season (March to September), but some additional water is needed to accomplish water demand in the winter season (October to February). The total volume of the missing water is 6993 m³. The daily water pumped, water drawn by the farmer and daily volume of the missing water in (m³/day) for all month of the year are shown in Table 12.

**Table 12:** Daily water pumped, water drawn and missing water of SPVWPS.

	Energy to operate pumping system (kWh)	Unused energy (kWh)	Average total head at water pump (meterW)	Volume of daily water pumped (m³/day)	Daily water drawn by farmer (m³/day)	Daily missing water (m³/day)
January	1381	0	60.74	125.48	125.16	59.03
February	1703	0	61.19	174.1	170.8	61.1
March	2443	42	61.83	256.2	229.58	0.0
April	2263	857	62.12	250.0	250.0	0.0
May	2209	1055	62.50	251.03	250.0	0.0
June	2180	948	62.20	244.8	250.0	0.0
July	2049	220	62.04	222.6	222.0	0.0
August	2266	49	62.11	244.5	244.2	0.0
September	2369	256	62.07	263.9	250.0	0.0
October	2142	22	61.51	244.51	244.3	4.8
November	1398	0	60.80	118.3	117.4	58.93
December	1097	0	60.47	70.8	70.8	49.58
Year	23502	3448	61.69	205.62	202.1	19.16

**Fig. 10:**
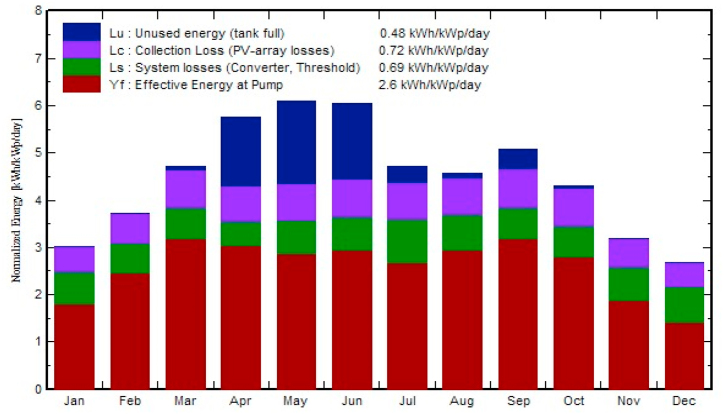
Normalized energy generation per kWp of proposed SPVWPS.

The normalized energy generation per kilowatt installed capacity of designed solar photovoltaic WPS is shown in Fig. 10. Normalized energy generation is higher in summer season (March to September) as compared to energy generation in winter season. The normalized values of the effective energy at water pump (Yf), system losses (converter, threshold etc.), collection losses (PV array losses) and unused energy (tank full) in the SPVWP system are 2.6 kW-hour/kilowatt-peak/day, 0.69 kW-hour/kilowatt-peak/day, 0.72 kW-hour/kilowatt-peak/day and 0.48 kW-hour/kilowatt-peak/day, respectively. As seen from the figure, the unused energy (Lu) of selected SPVWPS is minimum whereas collection losses (Lc) and system losses (Ls) seems to be slightly higher. This is because, SPV water pumping system (WPS) is designed to produce maximum amount of the water (m^3^) per day by using all of its available energy. When water production is at its peak, system faced major losses. However, if we reduce the volume of water pumped by the SPVWP system per day, we could minimize the system and collection losses, but at that time unused energy (Lu) in the designed system will be maximized.

**Fig. 11:**
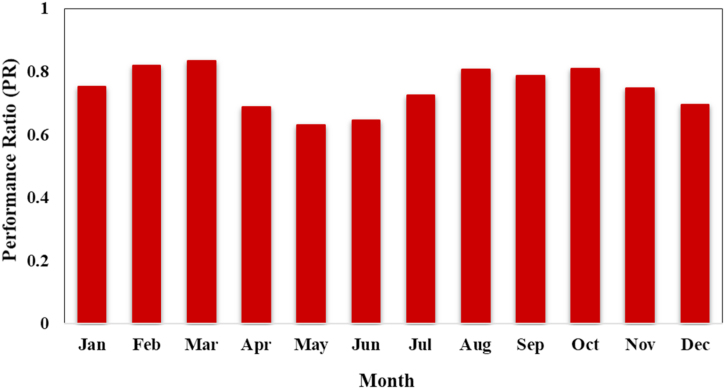
Performance ratio of the designed PV for WPS.

The monthly variation in performance ratio (PR) of the system are shown in Fig. 11. The PR is one of most crucial factors for determining the efficiency of photovoltaic (PV) system. The performance ratio (PR) is the ratio of actual energy generation to the theoretically achievable energy generation (Yf/Yr). Performance ratio is the photovoltaic system performance indicator that considers different environmental factors (irradiation, temperature etc.). For this reason, PR is used to compare the performance of photovoltaic energy systems that feed the utility grid around the world [39]. The performance ratio varies throughout the year. The PR in February, March, August and October was relatively high, and the PR in May, June and December was relatively low. The proposed photovoltaic (PV) system for WPS has an average annual PR of 74.62%. The high-performance ratio (PR) indicates that the SPV system for WPS will operate efficiently.

**Fig. 12:**
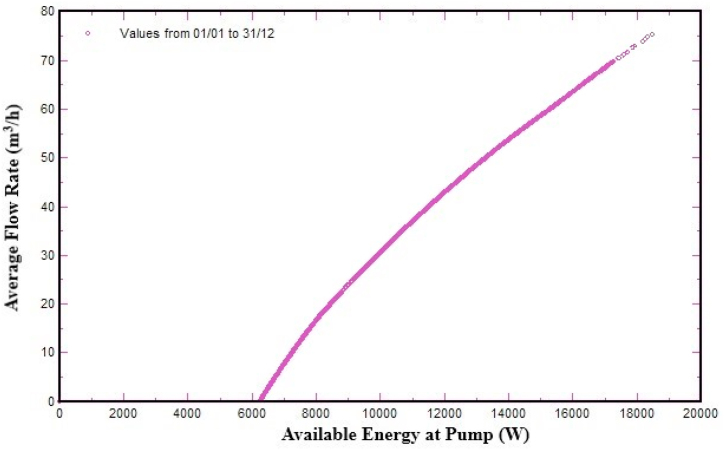
Flow-rate (m^3^/h) function of water pumps.

The relationship between the power (W) available to operate the pumping system and flow rate (m^3^/h) is shown in Fig. 12. When available power (W) at the water pump will be increased the flow rate (m^3^/h) of water pump will also be increased.

**Fig. 13:**
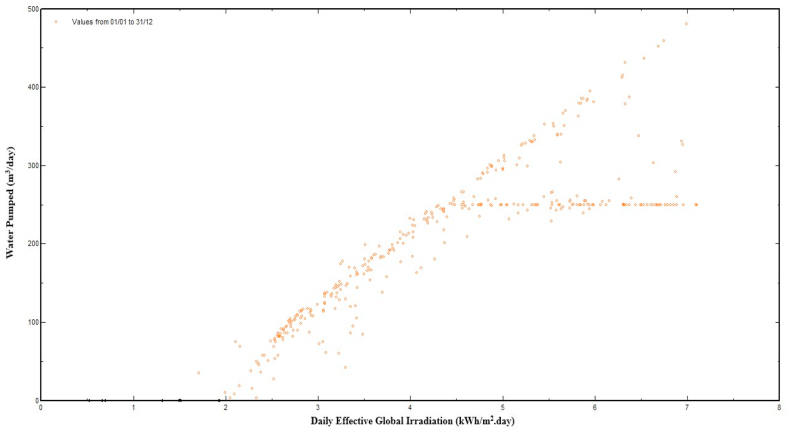
Daily water production versus effective global irradiation.

Fig. 12 shows the linear relationship between power (W) available to operate the pumping system and water flow rate (m^3^/h). Furthermore, the figure also demonstrates how available power affects the water flow rate and water pump size. If available power of the water pump is varying, the flow rate (m^3^/h) of the water pump will also be changed. As a result, the water pump controller (VFD) is very important for adjusting the flow rate and size of water pump in accordance with the available energy at the pump. It is observed that the solar PV water pumping system started to work at available power of 6100 W (6.1 kW) and below this power level, the water pump cannot work. At a power level of 8000 W, the flow rate of the water is 10 m^3^/h. When the available power from PV system is 10000 W (10 kW), the flow rate of water is 27 m^3^/h, and at the 1400 W energy level, the flow rate of the water is 55 m^3^/h. So, it is found that the flow rate of water is increased with increasing level of available power. Flow rate is zero below 6100 W and increases with the increase of available power, reaching its maximum value at noon, when production of PV system is at its peak level. The relationship between daily water productions with effective global irradiation are shown in Fig. 13.

From Fig. 13 it is observed that with the increase of solar irradiation, the water pump discharge (m^3^) increases. It is zero at the beginning of the day, water discharge increases with the increase of solar irradiation, reaching its maximum value at noon, and then gradually decreases in afternoon and it is zero in evening. The water discharge analysis in relation to solar irradiation shows that the water discharge increases with solar irradiation and becomes constant at a specific level of irradiation. When this particular solar irradiation level is reached, then there is no effect on water discharge.

**Fig. 14:**
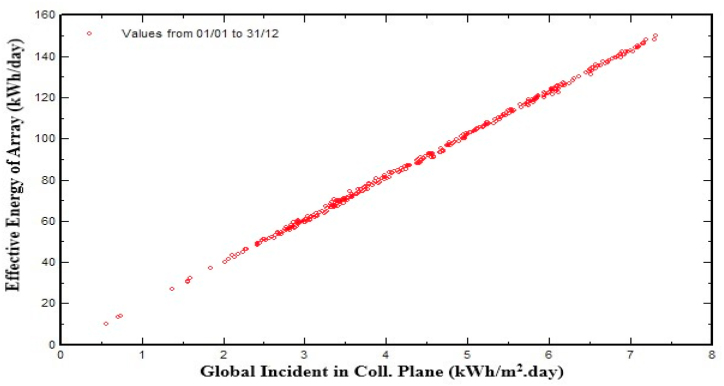
Daily input/output diagram of designed system.

It is also observed that the solar PV water pumping system started to work at a daily effective global irradiation level of 2 kWh/m^2^/day. Below this irradiation level, the water pump cannot work properly because the intensity of effective global irradiations is insufficient to supply the starting energy required to operate the PV-WPS. At an effective global irradiations level of 3 kWh/m^2^/day, the pump discharge is 100 m^3^/day. When the intensity of effective global irradiation is 3.5 kWh/m^2^/day, the pump discharge is 150 m^3^/day, and at the 4 kWh/m^2^/day irradiation level, the water pump discharge is 200 m^3^/day. The water discharge from the pump become constant (250 m^3^/day) after effective global irradiation level of 4.7 kWh/m^2^/day throughout the research study. The potential of effective global irradiation at research area is above 10 kW/m^2^/day in both season (summer and winter), while the SPV water pumping is operating at 4.7 kWh/m^2^/day. As a result, this SPVWPS met the water requirements of crops. Daily input/output diagram of designed system is shown in the Fig. 14.

Fig. 14 shows that effective energy (kWh) at output of photovoltaic array is directly related to the global incident in collector plane (kilowatt-hour/m^2^). As figure shows, the dot points are concentrated above 2.5 kWh/m^2^/day global incident in the collector plane. This scenario indicates that effective energy (kWh) of the photovoltaic (PV) array is 50 kWh/day or above for the most of time in the year.

#### 3.5.2 Losses in the pumping system

Table 13 represents the normalized losses in the water pumping system (WPS) for each month of the year. These energy losses are mostly caused by energy (kWh) losses under pump producing threshold and pump overload energy (kWh) (When the energy generated by photovoltaic system exceeds the maximum power of water pump). The energy losses under water pump producing threshold is 6392 kWh per year and these losses is maximum in month of June (715 kWh). Pump overload energy losses are zero for all months of the year because the system is perfectly designed to operate the water pump efficiently.

**Table 13:** Losses in the pumping system.

	PV array virtual energy at maximum power point (kWh)	Energy Losses under pump producing threshold (kWh)	Pump overload energy (kWh)	Available energy at water pump (kWh)	Energy under drawdown limit (kWh)	Unused Energy (kWh)	Energy to operate pumping system (kWh)
January	1902	521	0.000	1381	0.000	0	1381
February	2137	434	0.000	1703	0.000	0	1703
March	2987	502	0.000	2485	0.000	42	2443
April	3527	407	0.000	3120	0.000	857	2263
May	3849	584	0.000	3265	0.000	1055	2209
June	3700	573	0.000	3127	0.000	948	2180
July	2984	715	0.000	2269	0.000	220	2049
August	2893	578	0.000	2315	0.000	49	2266
September	3118	492	0.000	2626	0.000	256	2369
October	2669	505	0.000	2164	0.000	22	2142
November	1917	519	0.000	1398	0.000	0	1398
December	1658	561	0.000	1097	0.000	0	1097
Year	33342	6392	0.000	26950	0.000	3448	23502

### 3.6 SoSiT results

#### 3.6.1 Simulation input data

The SoSiT simulation input data are shown in Table 14.

**Table 14:** SoSiT simulation input data.

	Criterion	Value
1	Area	7 Zone (28 Acre)
2	Flowrate	14.2 LPS
3	Solar Power	24,750 Watt peak
4	Total Losses except Temperature	23%
	Soiling & Availability	6%
	Module Mismatch & Low Light	1%
	Manual Vs Auto Tracking	5%
	Cables	1%
	Inverter (Average)	5%
	Degradation @ 10 Y	5%
5	Manual tracked Installation	

#### 3.6.2 Threshold values

Threshold values are the passing criteria for system design as shown in Table 15. At least 3 criteria must be met for designing an optimal system.

**Table 15:** Threshold values.

Criterion	Threshold
Max Weekly Supply Gap % of Demand	<50%
Max Monthly supply Gap, % of Demand	<10%
Annual Supply Gap, m^3^	<25
Energy Efficiency: m^3^ potential/kWh prod.	<3.5

#### 3.6.3 Output parameters

The SoSiT model as a result calculates/determines the following decision parameters for each hour for the period of five years like expected output water of system, annual overcapacity/shortfall of the system, maximum weekly and monthly supply gap, annually water supply gap (CBM) and the water efficiency (CBM/kWh).

The results of the SoSiT simulation tool for 5 year are shown in the Table 16. The Maximum Weekly Supply Gap percentage of Demand (%) for all year is less than the Threshold value i.e < 50%. Similarly other passing criteria such as Maximum Monthly supply Gap percentage of Demand (%), Annual Supply Gap (m^3^) and Energy Efficiency (m^3^ potential/kWh) is also fall within Threshold values. Hence it is concluded that the system has passed all four criteria.

**Table 16:** SoSiT results.

	Year 1	Year 2	Year 3	Year 4	Year 5	Threshold
Max Weekly Gap	27%	40%	41%	38%	43%	<50%
Max Monthly Gap	0%	0%	0%	0%	0%	<10%
Annual Shortage (m^3^)	0	0	65	0	0	<25
Water Efficiency (m^3^/kWh)	3.2	3.2	3.2	3.2	3.2	<3.5

**Fig. 15:**
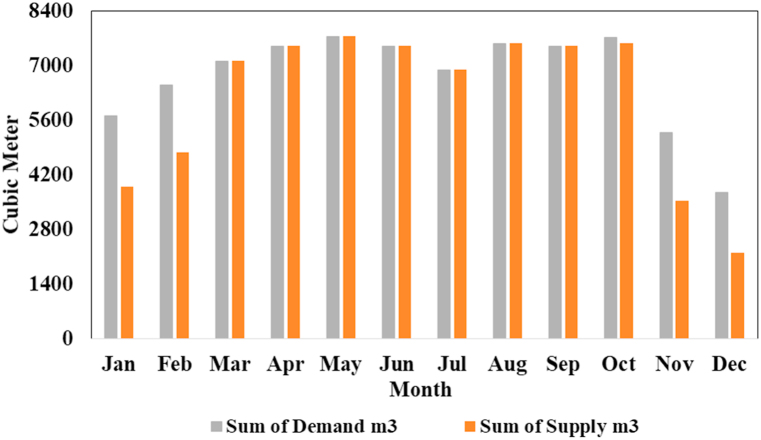
Demand and supply of Crop water by SPVWPS.

Actual water demand (m^3^) and supply (m^3^) by designed solar photovoltaic WPS are shown in Fig. 15. This indicates that the SPVWPS meets the desired water requirements of the crop throughout the year and is well matched. Especially during the summer season, SPVWPS fulfils the required water requirements. For example, in June and July, the total water demand is 7500 and 6883 cubic meters respectively, which is covered by the photovoltaic WPS. Similar to the findings of research studies by Raza et al. [21], Tamoor et al. [22] and Miran et al. [47], the photovoltaic WPS met the desired crop water requirements.

### 3.7 Socio-economic impact

#### 3.7.1 Cost analysis

The cost analysis aimed to calculate the total cost of the three systems powered by photovoltaic systems, diesel engine and electricity from utility grid, including total annual cost and total cost per hour. In order to ensure the project's long-term profitability and sustainability, it is also utilized to plan the unit (kWh) cost at which water should be sold. Once the simulation software has identified all possible technical solutions, first calculate the cost of each system. The variable part of the initial capital cost depends on the price of photovoltaic modules, diesel engine, water pumps, transportation and installation as well as the cost of pipes, cables, fittings, and structural components. The cost of the diesel engine, solar PV system, transformer, pumping unit, and other items is takes from local suppliers. Cost comparison of WPS operating on solar PV, diesel and electricity from national grid are shown in Table 17.

**Table 17:** Cost comparison of water pumping system (WPS) operating on solar PV, diesel and grid electricity.

Water pumping system cost breakdown	Solar PV	Diesel	Electricity from Grid
**Initial capital expenses**			
Cost of water pumping unit (€)	2,951	2,951	2,951
Electric Motor (€)	1,820	1,880	1,820
PV/Transformer (€)	12,739		2,060
System Housing (€)	544	544	544
Total initial capital expenses (P)	18,054	5,375	7,375
**Fixed cost (Parameter)**			
Estimated System Life (Years)	20	12	12
Salvage value, %	20	10	10
Interest rate (i), %	4	15	15
Taxes on P (%)	0	0	0
**Fixed Cost of the system**			
Depreciation including housing	722	403	553
Average Interest on investment	361	403	553
Taxes on water pumping operation	0	0	0
	0	0	0
Total annual fixed costs	1,083	806	1,106
**Variable cost (Parameters)**			
Average working hours/day	3	3	3
Unit cost of energy source utilized	0	0.6 €/L	0.054 €/kWh
Oil change after working hours	–	50	–
Lubricant cost	–	8.16	–
Number of Lubricant changes annually	–	22	–
Maintenance and Repair % of Capex	2%	4%	2%
Labour charges/hour	0.54	0.54	0.54
**Variable Cost of the system**			
Fuel/Electricity cost	0	1,971	441
Lubrication cost	0	179	0
Maintenance and Repair	271	215	148
Labour charges @ 74.39 €/month	0	0	0
Total annual variable cost	271	2,365	589
**Total Cost**			
Total cost per annum	1,354	3,171	1,695
Total cost per hour (€/h)	1.24	2.90	1.55
Unit cost (€/kWh)	0.17	0.39	0.21

**Fig. 16:**
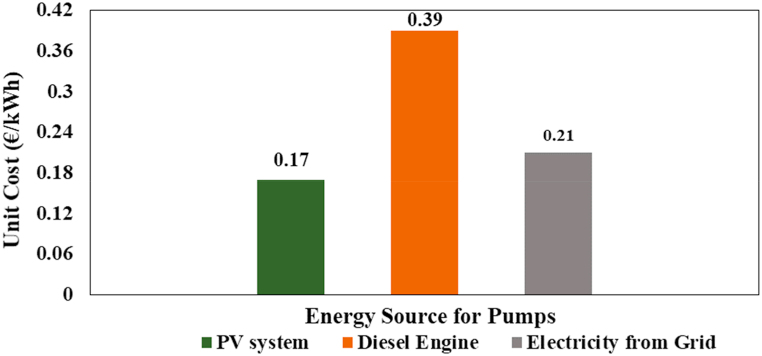
Unit cost of the solar, diesel and electricity from utility grid.

The initial capital cost of the diesel-powered WPS is lower than the solar photovoltaic WPS as shown in Table 17, but other costs of diesel-powered system are higher. In reverse, the initial capital cost of a solar photovoltaic WPS is higher, but the operational, maintenance and replacement cost is lower. Furthermore, photovoltaic systems do not require energy costs, so the annual variable cost of photovoltaic systems is much lower than a diesel-powered system. The photovoltaic system might be more suited for remote or rural areas because of its cheaper operational and maintenance cost, prolonged service life, and higher reliability. Diesel powered systems are more expensive to operate, maintain and now fuel prices are rising around the world, so these costs are continuing to increase. The unit (kWh) cost of the solar photovoltaic (PV) water pumping system (WPS) is 0.17 €/kWh, which is 56.41% and 19.04% less expensive than the cost of diesel and electricity from utility grid respectively as shown in Fig. 16.

#### 3.7.2 Industry development and farm level job creation

**Fig. 17:**
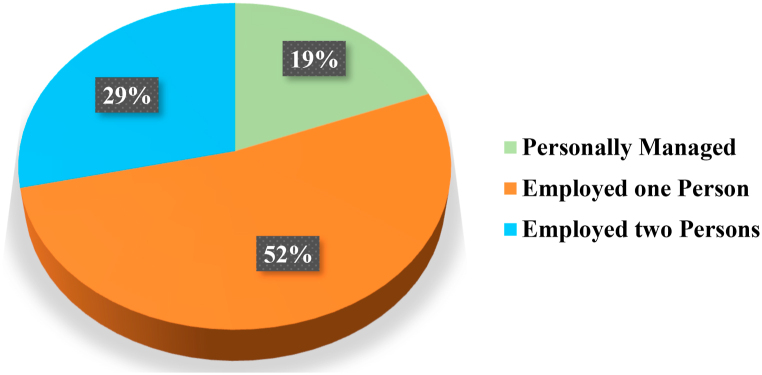
Personnel employed after installation of photovoltaic system.

Solar companies are hiring a significant number of engineers, management staff, and technicians. Many companies are working to install photovoltaic systems for WPS in targeted area. For the successful installation of the SPVWPS, companies hire qualified employees, including engineers (electrical and agricultural engineer), technicians have a diploma in the electrical or mechanical engineering, and administrative staff. It is expected that many jobs are created along with the growth of the renewable energy sector, which ensures environmental friendly and acceptable energy for the irrigated agriculture. For the operation of photovoltaic WPS, many additional jobs have been introduced in agriculture sector. The interview results showed that after installing the photovoltaic systems, 52% of the farms employed one person and 29% of the farms employed two people to operate the photovoltaic systems for WPS, as shown in Fig. 17.

#### 3.7.3 Farmer's satisfaction on PV systems to operate WPS

**Fig. 18:**
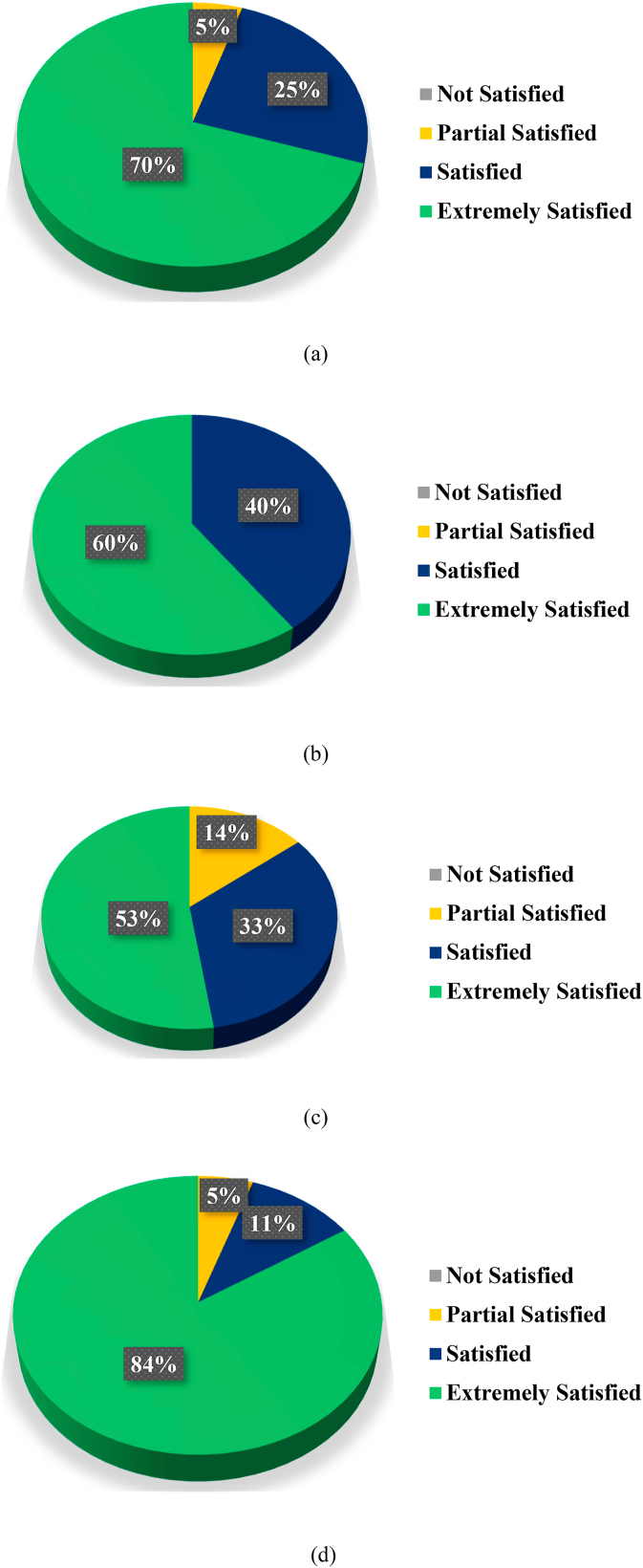
Famers Satisfaction on (a) on the operation of SPVWPS (b) on SPVWP system met water requirements of the crop (c) on yield using SPVWPS (d) operating cost.

Farmers are interviewed during the fieldwork in order to understand their satisfaction with the performance of the PV system used to operate water pumping system (WPS). In the questionnaire, the satisfaction of farmers is divided into 1 to 4 levels, where 1 and 4 correspond to "not satisfied" and "extremely satisfied," respectively. The results of the interviews with farmers showed that 70% and 25% of the farmers are extremely satisfied and satisfied, respectively on the operation of PV WPS as seen in Fig. 18 (a). They also gave it positive ratings and suggested it to other farmers. None of the farmers are "not satisfied". As shown in Fig. 18 (b), all farmers are extremely satisfied (60%) or satisfied (40%) that their irrigation system met water requirements of the crop. Furthermore, as shown in Fig. 18 (c), 53% of the farmers are "extremely satisfied" and claimed to be getting very high yields, while 33% claimed that their yields have increased and they are satisfied. In addition, 84% of farmers indicated that they did not incur any operating costs after the PV system was installed, as shown in Fig. 18 (d).

### 3.8 Discussion

In this research study, a modelling and performance analysis of solar photovoltaic WPS is presented by using PVSyst simulation software and mathematical analysis. Solar photovoltaic WPS has been optimally designed considering the daily water requirement and water resource details, solar resources, tilt angle and orientation, losses in PV and pumping system and performance ratio. The seasonal weather pattern varies from cold winters to sunny summers with peak temperatures of 31.09 °C. Approximately 60–75% of the annual rainfall occurs during the monsoon season from the month of July to September and there are an average 7 to 9 sunshine hours per day. The global horizontal irradiance (GHI) is maximum during the summer (April to June) and decreases during the winter season (November to February). The maximum GHI is in month of May (191.7 kWh/m^2^) and the minimum in the month of December (112.3 kWh/m^2^). The diffuse horizontal irradiance (DHI) is maximum in the month of June (100.8 kWh/m^2^) and minimum in the month of January (43.7 kWh/m^2^). Based on global horizontal irradiance data, not only solar photovoltaic WPS, but many other PV system are installed to fulfill the growing energy demand. The average peak ambient temperatures of the site are in the range of 29–32 °C, during the summer season (May to August) and the wind velocity during this period are in the range of 4.5–5.5 m/s. It has been observed that the efficiency of photovoltaic modules reduces as their temperature increases. The output power of the photovoltaic module is 450.1 W at the operating temperature of 25 °C and a solar irradiance level of 1000 W/m^2^, and the output power is 398.5 W at the operating temperature of 50 °C and a solar irradiance level of 1000 W/m^2^.

A comparative analysis of output energy produced by the PV system installed with fixed-mounted structure and single-axis solar tracker are carried out. In comparison to fixed-mounted structure, single-axis has been shown to be very efficient in terms of output energy. In this research work, a series of simulation experiments are performed in order to determine the optimum tilt angle for the photovoltaic array, which is the angle at which the PV system produces the maximum output energy at the proposed location. After evaluating the experimental results, it can be determined that photovoltaic (PV) energy production system installed at a tilt angle of 15° is more efficient as compared to photovoltaic systems installed at other tilt angles. The annual generation of the PV system is 33342 kWh at this tilt angle, while the annual energy generation at 3°, 7°, 22° and 30° are 32030 kWh, 32771 kWh, 30738 kWh and 27418 kWh respectively. The maximum output energy was produced in June (3849 kWh) and the minimum output energy was produced in December (1658 kWh). The energy wasted/consumed due to module array mismatch losses and ohmic wiring losses are 374.16 kWh and 298.83 kWh, respectively.

The annual PV array virtual energy at MPP of the designed photovoltaic system is 33342 kW-hour and the annual energy available to operate the WPS is 23502 kW-hours. The total annual water demand of the site is 80769 m³ and the total volume of water pumped is 75054 m³. The designed solar photovoltaic water pumping system can meet 92.93% of the irrigation water demand Normalized energy generation is higher in summer season (March to September) as compared to energy generation in winter season. The normalized values of the effective energy at the water pump (Yf), system losses (converter, threshold etc.), collection losses (PV array losses) and unused energy (tank full) in the SPVWP system are 2.6 kW/kWpeak/day, 0.69 kilo-watt/kWpeak/day, 0.72 kW/kWpeak/day and 0.48 kilo-watt/kWpeak/day, respectively. The annual average PR of the proposed PV system for WPS is 74.62%.

The linear relationship between available power at the pump (W) and pump flow rate show that the SPVWPS started to work at the available power of 6100 W (6.1 kW) and below this energy level, the water pump cannot work. At a power level of 8000 W, the flow rate of the water is 10 m^3^/h. When the available power from PV system is 10000 W (10 kW), the flow rate of the water is 27 m^3^/h, and at the 1400 W power level, the flow rate of the water is 55 m^3^/h. So, it is found that the flow rate of water is increased with increasing level of available energy.

Solar photovoltaic WPS contributes to the development of farmers. Farmers are interviewed during the fieldwork in order to understand their satisfaction with the performance of PV system used to operate water pumping system (WPS). The results of the interviews with farmers showed that 70% and 25% of the farmers are extremely satisfied and satisfied, respectively with the operation of PV WPS. They also gave it positive ratings and suggested it to other farmers. None of the farmers are "not satisfied". All farmers are extremely satisfied (60%) or satisfied (40%) that their irrigation system met the water requirements of the crop. Furthermore, 53% of the farmers are "extremely satisfied" and claimed to be getting very high yields, while 33% claimed that their yields have increased and they are satisfied. In addition, 84% of farmers indicated that they did not incur any operating costs after the PV system was installed. It is expected that many jobs are created along with the growth of the renewable energy sector, which ensures environmental friendly and acceptable energy for irrigated agriculture. The interview results showed that after installing the photovoltaic systems, 52% of the farms employed one person and 29% of the farms employed two people to operate the photovoltaic systems for WPS.

The unit (kWh) cost of solar photovoltaic (PV) water pumping system (WPS) is 0.17 €/kWh, which is 56.41% and 19.04% less expensive than the cost of diesel and electricity from the utility grid, respectively. The photovoltaic system might be more suited for remote or rural areas because of its cheaper operational and maintenance cost, prolonged service life, and higher reliability. Diesel powered systems are more expensive to operate, maintain, and now fuel prices are rising around the world, so these costs are continuing to increase. The modelling and performance analysis of the solar photovoltaic WPS is the most significant global validity of this research work. This research examines the performance of PV systems as well as the performance of solar photovoltaic WPS and provides better results than research conducted by some other researchers [47].

## 4 Conclusion

Research has shown that solar photovoltaic WPS can provide sustainable and climate-smart energy technologies for efficient irrigation system in rural areas those are not connected to the national electric grid. This can provide significant socioeconomic and environmental benefits. In water-scarce countries, primarily in desert regions, solar photovoltaic WPS can help to balance and increase crop yields, lessen the effects of drought, and overcome the pressure of water shortage during the dry seasons. In order to alleviate poverty in rural regions, solar photovoltaic WPS will stimulate the farmers to grow high value crops like vegetables and orchards. By introducing solar photovoltaic WPS in the agriculture sector, the dependence of agriculture on the electric grid is significantly reduced, farmers will no more depend on a limited power supply from the electric grid and they can receive clean and sustainable energy from PV systems. The annual PV array virtual energy at MPP of the designed photovoltaic system is 33342 kW-hour and the annual energy available to operate the WPS is 23502 kW-hours. The maximum output energy was produced in June (3849 kWh) and the minimum output energy was produced in December (1658 kWh). The energy wasted/consumed due to module array mismatch losses and ohmic wiring losses are 374.16 kWh and 298.83 kWh, respectively. The total annual water demand of the selected site is 80769 m³ and designed SPWPS pumped 75054 m³ of water, supplying 92.93% of the irrigation demand. Normalized energy generation is higher in summer season (March to September) as compared to energy generation in winter season. The normalized values of the effective energy at the water pump (Yf), system losses (converter, threshold etc.), collection losses (PV array losses) and unused energy (tank full) in the SPVWP system are 2.6 kW/kWpeak/day, 0.69 kilo-watt/kWpeak/day, 0.72 kilo-watt/kWpeak/day and 0.48 kilo-watt/kWpeak/day, respectively. The annual average PR of the proposed PV system for WPS is 74.62%. Solar photovoltaic WPS contributes to the development of farmers. Farmers are interviewed during the fieldwork in order to understand their satisfaction with the performance of the photovoltaic system used to operate the water pumping system (WPS). The results of the interviews with farmers showed that 70% of the farmers are extremely satisfied with the operation of PV WPS. All farmers are extremely satisfied (60%) or satisfied (40%) that their irrigation system met the water requirements of the crop. Furthermore, 53% of the farmers are "extremely satisfied" and claimed to be getting very high yields, and 84% of farmers indicated that they did not incur any operating costs. The interview results also showed that after installing the photovoltaic systems, 52% of the farms employed one person and 29% of the farms employed two people to operate the photovoltaic systems for WPS. The unit (kWh) cost of solar photovoltaic (PV) water pumping system (WPS) is 0.17 €/kWh, which is 56.41% and 19.04% less expensive than the cost of diesel and electricity from the utility grid, respectively. Overall installation costs are anticipated to decline over the next few years due to falling photovoltaic prices, and the area needed for a utility scale PV system is also predicted to decrease due to rapid technological advancement. By expanding the installed capacity of PV system, farmers with larger land holdings can get a faster return on investment. Moreover, by using a bidirectional meter, they can export any excess energy to the utility grid to receive additional financial support or revenue. SDG-2 sets the goal of doubling agricultural crop yield and incomes of small-scale farmers of producers by 2030. As fuel prices continue to increase, solar photovoltaic WPS is a suitable option for consumers. Farmers can meet their electricity needs self-sufficiently, thus increasing the contributions of renewable energy in society. In this way, the goals of reaching net-zero by 2070 and increasing farmer income are achieved. The energy needs of agriculture in the future will be met through sustainable and clean energy SPVWPS, thereby achieving SDG-7. Furthermore, the results of this study can also assist investors, engineers and designers in developing a plan for the designing and installation of solar photovoltaic WPS in any location in the world.

## Author contribution statement

Salman Habib, Haoming Liu, Muhammad Tamoor, Muhammad Ans Zaka, Youwei Jia: Conceived and designed the experiments; Performed the experiments; Analyzed and interpreted the data; Contributed reagents, materials, analysis tools or data; Wrote the paper.

Abdelazim Hussien, Hossam Zawbaa, Salah Kamel: Analyzed and interpreted the data; Contributed reagents, materials, analysis tools or data; Wrote the paper.

## Data availability statement

Data will be made available on request.

## Declaration of competing interest

The authors declare that they have no known competing financial interests or personal relationships that could have appeared to influence the work reported in this paper.
