# Supplementary material for: Technical modelling of solar photovoltaic water pumping system and evaluation of system performance and their socio-economic impact
Source: Heliyon. 2023 May 8;9(5):e16105. doi: 10.1016/j.heliyon.2023.e16105 (PMC10205495; doi:10.1016/j.heliyon.2023.e16105)
Supplement: Multimedia component 1 [file mmc1.pdf]

Evaluation Survey

Survey Date:__________________________

A. Farmer Details:

1. Name of Farmer: ______________________________
2. Cell No.: ______________________________
3. Village/Mouza: ______________________________
4. Tehsil: ______________________________
5. District: ______________________________
6. Division: ______________________________
7. Total land holding (acres): ______________________________

   a. Self-owned Land (acres):________________b) Rented-in Land (acres):__________________________

8. Irrigation Practice (SPVWPS/Flood): ______________________________

9. Area under SPVWPS/Flood (acres) for current study: ______________________________

B. Site Details (Under Study):

1. GPS Co-ordinates: N:________________________________E: ________________________________

2. Year of SPVWPS installation (e.g. 2020): ______________________________

3. Water table depth (ft): ______________________________

4. Water source (Canal/Ground water/Canal+Ground water): ______________________________

5. Energy Source for Irrigation (Electricity/Diesel/Solar): ______________________________

6. Grid Connection Availability at Site (Yes/No): ______________________________

7. Distance from nearby transformer (m): ______________________________

8. Capacity of power source for Irrigation (hp): ______________________________

9. Design discharge of SPVWPS/TW (Lps): ______________________________

10. Sanctioned discharge of canal water (Lps): ______________________________

C. Farmer’s Satisfaction and Feedback:

i. Industry Development and Farm Level Job Creation

1. How many persons / labor are employed after installing HEIS (No. of Persons): ____________________________

ii. Farmer’s Satisfaction on PV Systems to operate WPS

Farmer satisfaction (1 to 4) (1 = Not satisfied, 4 = Extremely satisfied)

1. Are you satisfied regarding Solar PV + WPS fulfilling crop water needs? ________________________________
2. Are you getting good yields using Solar PV + WPS? ____
3. Are you satisfied with Solar PV + WPS operations? ____
4. Are you satisfied with cost of operating Solar PV + WPS? ____

   ----

5. Any Suggestion for Improvement? ____

D. Cropping pattern (Crops/Orchards sown throughout the year):

Crop	Area of crop (acres)	Under SPVWPS (Yes/No)	Average Yield (kg/acre)	Total Costs /season (PKR/acre)	Total Sales / season (PKR/acre)

Orchards (Fruits)	Area of Orchard (acres)	Age of Orchard (Months)	Under SPVWPS (Yes/No)	Average Yield (kg/acre)	Total Costs /season (PKR/acre)	Total Sales / season (PKR/acre)

E. Irrigation practice (Farmer Feedback; Fill-in the relevant Energy Source only):

Month	SPVWPS Installed (Yes/No)	Operation Time (minutes)	Actual Operating Frequency (days)	Actual Canal availability (minutes)	Diesel Consumption (Litres/hr)	Electricity Bill (PKR)	Solar (PV)	R&M + Other Costs (PKR)
January								
February								
March								
April								
May								
June								
July								
August								
September								
October								
November								
December								

Hint for frequency: Daily irrigation=1, Irrigation on alternate day=2, Irrigation every 3rd day=3, Irrigation every 4th days=4, Irrigation every 5th days=5 and so on
