# Supplementary material for: Technical modelling of solar photovoltaic water pumping system and evaluation of system performance and their socio-economic impact
Source: Heliyon. 2023 May 8;9(5):e16105. doi: 10.1016/j.heliyon.2023.e16105 (PMC10205495; doi:10.1016/j.heliyon.2023.e16105)
Supplement: Multimedia component 2 [file mmc2.pdf]

Form to confirm authorship changes for *Heliyon*

This form must be ***signed by all authors*** when there is a change in authorship which includes changes to any of the following items: author name(s), order of the authors, the corresponding author(s), the addition of authors, the removal of authors and changes in affiliation.

By personally signing this note, **all** authors confirm that: I) the changes are in accordance with their scientific contribution, II) they agree with all the changes and III) confirm that the authorship list conforms to the authorship criteria outlined on *Heliyon's* ethics page. IV) it is the responsibility of the corresponding author to get the signature from all co-authors accepting the change. In case of any ethic violation/malpractice in the signature, the corresponding author is accountable. The completed form should be returned along with the final/revised manuscript to proceed further with the manuscript. Manuscripts for which incomplete forms have been submitted will be rejected within 5 working days.

Any disputes on the authorship list and contributions need to be resolved by the involved scientists and *Heliyon* will only proceed with the evaluation of the manuscript once we receive confirmation, through this form, that such an agreement between the authors has been reached.

Manuscript number: HELIYON--D-23-01521R1

Article title: Technical Modelling of Solar Photovoltaic Water Pumping System and Evaluation of System Performance and Their Socio-Economic Impact

Complete new author list: **Salman Habib, Haoming Liu, Muhammad Tamoor, Muhammad Ans Zaka, Youwei Jia, Abdelazim G. Hussien, Hossam M. Zawbaa, Salah Kamel**

Date: April 30, 2023

#	First name	Last name	Order change (Y/N)	Addition / Deletion	Change in Author name (Y/N)	Affiliation Change (Y/N)	Reason for the change	Signature
1	SALMAN	HABIB	N	N	N	Y	One new affiliation is added for this author due to contractual issue with both institutions	[signature]

2	Haoming	Liu	N	N	N	N	/	[illegible]
3	Muhammad	Tamoor	N	N	N	N	/	[illegible]
4	Muhammad Ans	Zaka	N	N	N	N	/	[illegible]
5	Youwei	Jia	N	Y	N	N	1) Conceived and designed the experiments<br>2) Analyzed and Interpreted the data<br>3) "wrote the paper"	[illegible]
6	Abdelazim	G. Hussien	N	Y	N	N	1) Performed the experiments<br>2) Contributed reagent, materials, analysis tools or data	A. Azim
7	Hossam	M. Zawbaa	Y	N	N	Y	Order is changed due to addition of new author. One of the affiliations is changed due to the contractual issues.	Houssam
8	Salah	Kamel	Y	N	N	N	Order is changed due to addition of new author.	[illegible]
